# Associations of Transcription Factor 21 Gene Polymorphisms with the Growth and Body Composition Traits in Broilers

**DOI:** 10.3390/ani12030393

**Published:** 2022-02-08

**Authors:** Linyong Shen, Jiaqiang Yu, Yaowen Ge, Hui Li, Yumao Li, Zhiping Cao, Peng Luan, Fan Xiao, Haihe Gao, Hui Zhang

**Affiliations:** 1 Key Laboratory of Chicken Genetics and Breeding, Ministry of Agriculture and Rural Affairs, Key Laboratory of Animal Genetics, Breeding and Reproduction, Education Department of Heilongjiang Province, College of Animal Science and Technology, Northeast Agricultural University, Harbin 150030, China; sly18846814837@163.com (L.S.); geyaowen1998@163.com (Y.G.); lihui@neau.edu.cn (H.L.); liyumao@neau.edu.cn (Y.L.); caozhipingneau@126.com (Z.C.); a47293768@126.com (P.L.); 2 Forest Investigating and Planning Institute of Daxinganling, Yakshi 022150, China; yujiaqiang5259@163.com; 3 Fujian Sunnzer Biotechnology Development Co., Ltd., Nanping 354100, China; snxf@sunnercn.com (F.X.); snghh@sunnercn.com (H.G.)

**Keywords:** single nucleotide polymorphism, marker-assisted selection, broiler chicken

## Abstract

**Simple Summary:**

The functional SNPs discovered in this work will give helpful information on the crucial molecular markers that may be employed in breeding efforts to improve the heart development of broiler chickens.

**Abstract:**

This study aims to identify molecular marker loci that could be applied in broiler breeding programs. In this study, we used public databases to locate the *Transcription factor 21* (*TCF21*) gene that affected the economically important traits in broilers. Ten single nucleotide polymorphisms were detected in the *TCF21* gene by monoclonal sequencing. The polymorphisms of these 10 SNPs in the *TCF21* gene were significantly associated (*p* < 0.05) with multiple growth and body composition traits. Furthermore, the *TT* genotype of g.-911T>G was identified to significantly increase the heart weight trait without affecting the negative traits, such as abdominal fat and reproduction by multiple methods. Thus, it was speculated that the g.-911T>G identified in the *TCF21* gene might be used in marker-assisted selection in the broiler breeding program.

## 1. Introduction

Chicken (*Gallus gallus*) is the most commonly raised poultry by humans. Since the 1950s, the growth rates and meat yield of broilers have significantly improved. However, with the rapid growth of broilers, some problems inducing huge economic losses have emerged, such as obesity, ascites syndrome, leg diseases, broiler immunity decline, and sudden death [1]. The growth rate of broilers is positively correlated with these unfavorable traits. As a result, it is difficult to simultaneously increase the growth rate and decrease these unfavorable traits in broilers by the traditional phenotypic selection method alone. Notably, molecular marker-assisted selection (MAS) can provide new ideas for overcoming such problems [2]. The combination of molecular genetic marker breeding with traditional phenotypic selection helps to greatly improve the breeding efficiency and accelerate the breeding process [3]. In recent years, with the rapid development of molecular genetics, genetic markers have been gradually applied in the MAS of livestock and poultry [4]. This technology contributes to substantially improving the breeding efficiency and shortening the generation intervals [5]. Among the numerous molecular markers, single nucleotide polymorphisms (SNPs) have been the most extensively studied [6,7,8,9]. Therefore, SNPs are of practical significance to identify genes or markers related to the economically important traits of broilers.

Transcription factor 21 *(TCF21)* plays an important role in a variety of economically important traits such as heart development [10], testis formation [11], and adipogenesis [12] in chickens. This study aims to identify the SNPs of the *TCF21* gene that are significantly associated with the growth and body composition traits of broilers. The results of this study can provide useful information for the molecular genetic marker-assisted breeding of the economically important traits of broilers.

## 2. Material and Methods

### 2.1. ChIP-Seq Analysis

The ChIP-seq dataset for histone modification marks (H3K4me3, H3K27ac, H3K4me1, H3K27me3) and CTCF data in the seven tissues of chickens used in this work were downloaded from the GEO Datasets: GSE158430 [13]. The Bedtools software 2.29.1 version was used to separate the ChIP-seq datasets of all tissues within the same merged [14]. The reference genome and annotation file for galGal6 (*Gallus gallus*) were downloaded from the UCSC Genome Browser (http://hgdownload.soe.ucsc.edu/goldenPath/galGal6/bigZips/, accessed on 16 June 2021). These combined data were genetically annotated using the ChIPseeker software 1.30.2 version [15] and visualized using the IGV browser (http://software.broadinstitute.org/software/igv/, accessed on 16 June 2021).

### 2.2. Experimental Populations and Phenotype Measurements

Details on the Northeast Agricultural University broiler lines divergently selected for abdominal fat content (NEAUHLF) were described by Zhang et al. [16]. In this study, altogether, 675 male birds from the generations 21 (G21) populations of NEAUHLF were used for an association study. All birds were raised and managed in accordance with the routine commercial broiler feeding procedures.

For the G21 populations, the body weight (BW) of all male birds was measured at 1, 3, 5, and 7 weeks of age (assigned as BW1, BW3, BW5, and BW7, respectively). At the age of 7 weeks, the above birds were slaughtered, and the body composition traits were recorded. Before slaughter, the chest angle (ChA), keel length (KeL), body oblique length (BoL), chest width (ChW), metatarsus length (MeL), and metatarsus circumference (MeC) of all birds were measured. After slaughter, the carcass weight (CW), abdominal fat weight (AFW), liver weight (LW), muscular stomach weight (MSW), glandular stomach weight (GSW), heart weight (HW), spleen weight (SW), and testicle weight (TeW) were measured. For the reporting of results, we complied with the Animal Research: Reporting In Vivo Experiments (ARRIVE) guidelines [17].

### 2.3. Genotyping of SNPs

In total, 20 individuals from NEAUHLF were randomly selected (with 10 birds from each line). Then, the whole gene of *TCF21*, including the gene body region, the 5 ‘flanking region (2000 bp), and the 3’ flanking region (2000 bp), was sequenced, which resulted in 10 SNPs, referred to as g.-1243C>T, g.-1171T>C, g.-911T>G, g.-891C>T, g.691C>T, g.897T>C, g.1033G>A, g.1892A>G, g.2091C>T, and g.2155C>T, according to their respective positions in the *TCF21* gene.

Using Primer Premier 5.0 (Premier, Canada), a series of PCR primers were designed to amplify the various portions of the chicken *TCF21* genomic DNA sequence based on the chicken gene sequence (NCBI Reference Sequence: NC_006090.5), and all PCR primer sequences were synthesized and purified by Invitrogen (Camarillo, CA, USA). The primer sequences are shown in Table 1.

Furthermore, the total genomic DNA was extracted from 675 male birds of the G21 of NEAUHLF for PCR analysis, according to previous depiction [18]. These SNPs were genotyped with the PCR-restriction fragment length polymorphism (PCR-RFLP) method. The PCR amplification system included: 50 μg/μL genomic DNA 1 μL, 10 mmol/L dNTP 0.8 μL, 10 × PCR Buffer 1 μL, 10 mol/L upstream and downstream primers each 0.2 μL, 5 U/μL Taq DNA polymerase 0.1 μL, and deionized water 6.7 μL. The PCR amplification conditions were as follows: pre-denaturation conditions were all 94 °C for 5 min, denaturation conditions were all 94 °C for 30 s, extension conditions were all 72 °C for 30 s, and ultimate extension conditions were all 72 °C for 7 min. The annealing conditions and cycle number are listed in Table 1. After the PCR reaction was finished, 1.2% of the agarose gel was configured, the PCR amplification products were added, and the electrophoresis time was set for 20 min at 100 V. This agarose gel was removed from the electrophoresis solution and placed in the gel imaging analysis system to take pictures for identification. All the PCR reagents and electrophoresis reagents were obtained from Dalian Treasure Biological Engineering Co., Ltd. (Dalian, China).

The PCR amplification product was detected by the agarose electrophoresis of the target band single bright sample, and carried out by an enzymatic reaction test and enzymatic reaction system (2 μL of PCR product, 1 μL of Cutsmart Buffer, 6.8 μL of deionized water, and 0.2 μL of endonuclease, which were digested overnight at 37 °C). The SnapGene 5.0 Viewer (https://www.snapgene.com/snapgene-viewer/, accessed on 20 October 2021) was used to select the restriction enzymes, and the endonuclease for each SNP are displayed in Table 1. New England Biolabs provided all of the restriction enzymes (New England Biolabs, Ipswich, MA, USA). The digested products were detected by 3.0% agarose gel electrophoresis at 110 V for 50 min, and three genotypes were acquired for each of the 10 SNPs (Figure S1).

### 2.4. Transcription Factor Binding Site Analysis

To explore the potential molecular mechanisms underlying the association of SNPs loci in the *TCF21* gene with the economically important traits in broiler chickens, bioinformatic analysis was performed using three transcription factor binding site software, including JASPAR (http://jaspar.binf.ku.dk/, accessed on 12 December 2021), TFBIND (http://tfbind.hgc.jp/, accessed on 12 December 2021), and Mulan (http://mulan.dcode.org/, accessed on 12 December 2021). These three bioinformatics software predicted overlapping transcription factors that were considered to possibly bind to the DNA sequence of SNPs in the *TCF21* gene.

### 2.5. Statistical Analyses

The difference in allele frequencies between the lean and fat lines was determined and examined using the Chisquare test, with *p* < 0.05 as a significant difference between the lean and fat lines.

In this study, the JMP 7.0 software (SAS Inst. Inc., Cary, NC, USA) was employed for establishing a generalized linear mixed model to analyze the associations of SNP polymorphisms with the growth and body composition traits, with *p* < 0.05 being adopted as a threshold. In addition, the significant differences between the least-square means of different genotypes were calculated by the contrast test (*p* < 0.05). Then, the statistical model for analyzing the associations of genotypes with the growth and body composition traits was constructed based on the population characteristics [19]. The following model was utilized:

Y = μ + G +L + F (L) + D (F, L) + BW7 + e **I**

where Y is the observed value of traits, μ stands for the population mean, G indicates the genotype fixed effect, and L suggests the line fixed effect. In addition, F (L) indicates the random effect of the family within the line, whereas D (F, L) represents the random effect of dams in the family of the line, and e is the random effect. Model **I** was adopted to analyze the associations of SNP polymorphisms with the growth and body composition traits in 675 male birds (335 individuals from the lean line and 340 individuals from the fat line) from the G21 population of NEAUHLF, in which each line consisted of 40 family lines (one sire and four dams, respectively).

The statistical analysis model for genetic parameter estimation is shown below:

y = Xβ + Zu + e **II**

where y stands for the n-dimensional vector of the broiler growth and body composition traits, X represents the n × p matrix of fixed effects, β indicates the p-dimensional vector of fixed effects, Z suggests the n × q matrix of random effects, while u is the q-dimensional vector of random genetic effects, and e denotes the n-dimensional vector of random residual effects. Moreover, model **II** was applied in estimating the genetic parameters of the growth and body composition traits of the lean and fat lines in the G21 population of NEAUHLF.

## 3. Results

### 3.1. Identification of Genes Associated with Growth and Body Composition Traits in Broilers

**Figure 1:**
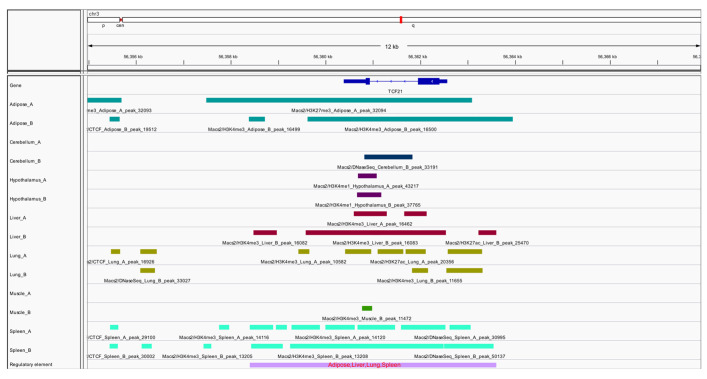
Bioinformatics analysis of the *TCF21* gene in chickens.

Genome-wide searches for genes affecting the important economic traits in broilers were conducted using the ChIP-seq data for histone modifications. The results revealed that the *TCF21* gene plays an important role in the adipose, liver, lung, and spleen tissues of broilers (Figure 1). Then, the entire gene of *TCF21*, as well as 2000 bp upstream and downstream of the *TCF21* gene, was sequenced, and 10 SNPs were identified (Figure S1).

The genotype frequencies and allele frequencies of those 10 SNPs in the *TCF21* gene in NEAUHLF were analyzed. Meanwhile, the chi-square independence test was conducted to calculate the differences in allele frequencies between the lean and fat lines. As discovered from the results, differences in the allele frequencies of these 10 SNPs were statistically significant between the lean and fat lines (*p* < 0.05; Table 2).

### 3.2. NEAUHLF Is an Ideal Test Material for Studying the Correlation between Growth and Body Composition Traits in Broilers

**Figure 2:**
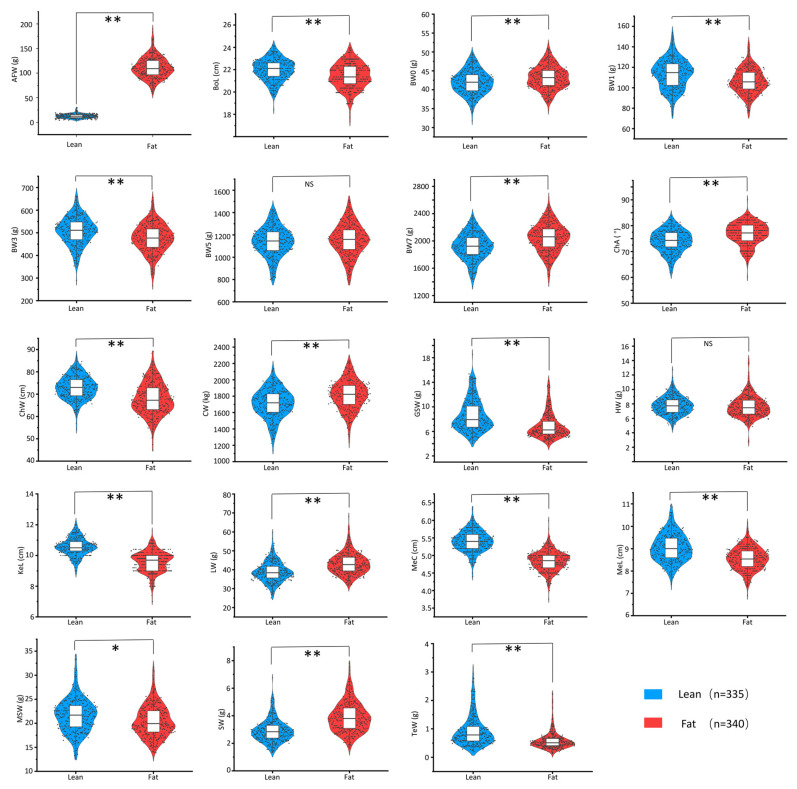
Phenotypic information regarding the growth and body composition traits in NEAUHLF. *, *p* < 0.05; **, *p* < 0.01; ns, *p* > 0.05 (nonsignificant). Abdominal fat weight (AFW), body oblique length (BoL), body weight at birth of age (BW0), body weight at 1 week of age (BW1), body weight at 3 weeks of age (BW3), body weight at 5 weeks of age (BW5), body weight at 7 weeks of age (BW7), chest angle (ChA), chest width (ChW), carcass weight (CW), glandular stomach weight (GSW), heart weight (HW), keel length (KeL), liver weight (LW), metatarsus circumference (MeC), metatarsus length (MeL), muscular stomach weight (MSW), spleen weight (SW), and testicle weight (TeW).

The phenotypic information of the growth and body composition traits is displayed in Figure 2. As observed from Figure 2, differences in most of these traits (except for HW and BW5) were significant (*p* < 0.05) between the lean and fat lines in the NEAUHLF population.

Furthermore, the heritability (h^2^) values of the growth and body composition traits were estimated. The results indicated that AFW, BW1, BW5, GSW, MSW, and TeW showed high heritability values (h^2^ > 0.3), whereas BW3, BW7, CW, and MeC had moderate values (0.2 < h^2^ < 0.3), and BoL, ChA, ChW, HW, KeL, LW, and MeL had low values (h^2^ < 0.2; Table 3). In addition, this study also estimated the genetic correlation (rg) between AFW and the other growth and body composition traits. As a result, at the genetic level, AFW was highly positively correlated (rg = 0.696 ± 0.223) with LW, but highly negatively correlated (−0.8 < rg < −0.3) with BoL, BW1, 3, 5, 7, GSW, KeL, and MeL. In addition, AFW showed low genetic correlations with ChW, CW, HW, MeC, MSW, and TeW (−0.3 < rg < 0.3; Table 3).

### 3.3. Associations of TCF21 Gene Polymorphisms with Growth and Body Composition Traits

**Figure 3:**
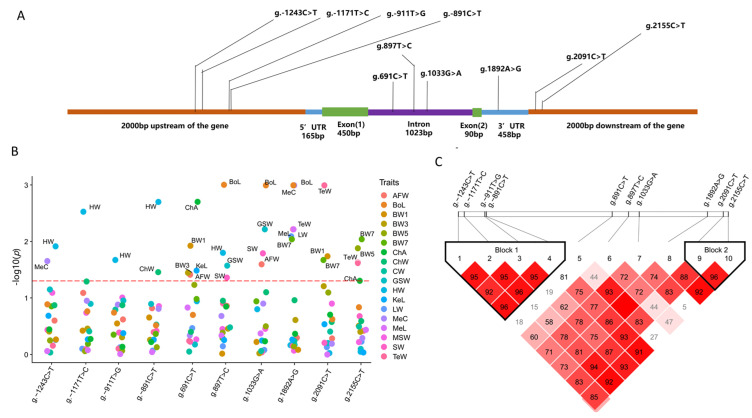
Identification of functional SNPs in the *TCF21* gene. (**A**) The distribution of SNPs in the *TCF21* gene. (**B**) Associations of *TCF21* gene polymorphisms with the growth and body composition traits in G21 populations. The red line indicates the significant threshold *p* < 0.05. (**C**) Linkage disequilibrium (LD) analysis of these 10 SNPs.

The positions of these 10 SNPs in the *TCF21* gene are shown in Figure 3A. Furthermore, this study analyzed the associations of the polymorphisms of those 10 SNPs in the *TCF21* gene with the growth and body composition traits in NEAUHLF. According to the results, the polymorphisms of g.-1243C>T, g.-1171T>C, g.-911T>G, and g.-891C>T were significantly related (*p* < 0.05) to HW. In addition, the polymorphisms of g.2091C>T and g.2155C>T were significantly correlated (*p* < 0.05) with BW and TeW (Figure 3B). Linkage disequilibrium (LD) analysis revealed the existence of 2 different LD blocks, with 4 SNPs from block 1 (g.-1243C>T, g.-1171T>C, g.-911T>G and g.-891C>T) in a strong linkage disequilibrium and 2 SNPs from block 2 (g.2091C>T and g.2155C>T) were also in a strong linkage disequilibrium state (Figure 3C). All these results suggest that SNPs within Block 1 may have important effects on the HW trait, and SNPs within Block 2 may have important effects on the TeW and BW traits.

**Figure 4:**
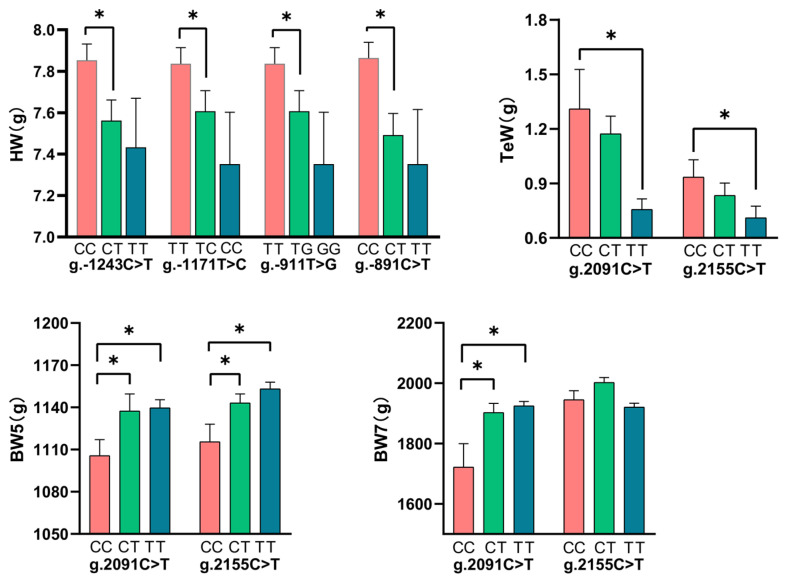
Analysis of the phenotypes of different genotypes of functional SNPs. * indicates a significant difference, *p* < 0.05.

Subsequently, this study further compared the least squares means of SNPs within these two blocks for different genotypes and traits. The results showed that the *CC* genotype of g.-1243C>T, *TT* genotype of g.-1171T>C, *TT* genotype of g.-911T>G, and *CC* genotype of g.-891C>T had higher heart weight than the heterozygous genotype (*p* < 0.05, Figure 4). Furthermore, the *TT* genotype of g.2091 C>T and g.2155C>T had higher body weight and lower testicle weight in broilers (*p* < 0.05; Figure 4).

In order to investigate the potential molecular mechanism underlying the association of the HW trait with four SNPs from Block 1 (g.-1243C>T, g.-1171T>C, g.-911T>G, and g.-891C>T), we carried out an in silico analysis of the transcription factor binding site using three bioinformatic tools. The results showed that g.-911T>G was located in multiple potential transcription factor binding regions (Table 4).

## 4. Discussion

In this study, two broiler lines were divergently selected for abdominal fat content for over twenty generations. The results revealed significantly different AFW values between the lean and fat lines. In addition to AFW, some other growth and body composition traits (except for HW and BW5) also showed significant differences (*p* < 0.05) between the lean and fat lines. The above results indicated that when AFW was selected, the other traits associated with AFW were also under selection. Therefore, the genetic correlations between AFW and other growth and body composition traits were estimated. The results indicated that AFW showed high genetic correlations with most of the other growth and body composition traits, including LW, GSW, BW1, 3, 5, 7, MeL, KeL, and BoL. Some studies also analyzed the correlations of AFW with the growth and body composition traits in chickens and reported that AFW exhibits high genetic correlations with BW5, BW7, LW, CW, and skin weight [26,27], which are consistent with our results. Therefore, the lean and fat lines were the ideal experimental materials used to study the growth and body composition traits.

It was discovered in this study that the polymorphisms of g.2091C>T and g.2155C>T in the *TCF21* gene were significantly associated (*p* < 0.05) with the TeW and BW traits. As revealed by studies on mammals, the *TCF21* gene plays an important role in the functions of testicles [11]. In addition, the testis growth and development of chickens are controlled by genetic factors [28,29,30,31,32], and cocks with lower TeW are usually less fertile [33]. Furthermore, it is found that male mice with the *TCF21* gene knockout have sex differentiation phenotypes [34]. The male sex determining factor *SRY* affects TeW through regulating *TCF21* [35,36]. Regrettably, the least squares mean analysis revealed that the *TT* genotype of g.2091C>T and g.2155C>T had higher body weight and lower testicle weight. It also indicated that selection for these two SNPs did not result in neither fast growth rate (BW) nor high reproductive performance (TeW) in broilers.

Heart hypertrophy increases the risk of sudden death in broilers, especially those with higher BW and AFW traits [37]. This research study found that the polymorphisms of g.-1243C>T, g.-1171T>C, g.-911T>G, and g.-891C>T were significantly related (*p* < 0.05) to the HW trait. Some literature reports 13 susceptible sites are detected in a GWAS on human coronary heart disease, among which rs12190287 is located at the 3’UTR of *TCF21* [38,39]. Generally, *TCF21* is expressed in mesoderm cells in the epicardial organ and then in mesenchymal cells that form the pericardium [40]. The loss of *TCF21* in chickens leads to epicardial blistering, increased smooth muscle differentiation on the heart surface, a paucity of interstitial fibroblasts, along with neonatal lethality [10]. It is encouraging that the least squares mean analysis revealed that the *CC* genotype of g.-1243C>T, *TT* genotype of g.-1171T>C, *TT* genotype of g.-911T>G, and *CC* genotype of g.-891C>T had higher heart weight (*p* < 0.05; Figure 4). It also indicated that selection for these four SNPs could improve the HeW trait without affecting other unfavorable traits at the same time in broilers. The non-coding regions of genes have a large number of regulatory elements, including enhancers, promoters, and silencers. Studies have shown that SNP located within these regulatory elements can affect traits by influencing the activity of regulatory elements [41,42,43]. In silico analysis suggested that the g.-1243C>T was located in the regions of potential binding of *BACH1* [20], and the g.-911T>G was located in multiple transcription factor binding regions (*GATA4, SMAD1*, and *SOX17*). Studies have shown that these transcription factors all play important roles in animal heart development [21,22,23,24,25]. It is hypothesized that the *TCF21* gene g.-911T>G regulates the HW trait probably through binding to transcription factors (*GATA4*, *SMAD1*, and *SOX17*) to influence the activity of regulatory elements in this region.

## 5. Conclusions

In this study, the associations of *TCF21* gene polymorphisms with the growth and body composition traits in broilers were analyzed. The results indicate that the g.-911T>G in the *TCF21* gene may be important molecular markers that affect the HW trait, and could be used in breeding programs to improve the heart development of broilers.

## Figures and Tables

**Table 1:** Primers used to amplify the SNPs in the *TCF21* gene.

SNP ID	Primers (5′ to 3′)	Size of Product (bp)	Annealing Conditions	Number of Cyclesr	Endonuclease
g.-1243C>T (rs741031208)	F: CTGAATAGTTGGATTTTCCCCTGCC R:CTGATGGAGTCGAAGAGGGGTTTTA	389	61 °C 35 s	35	*HPy*CH4IV
g.-1171T>G (rs312348545)	F:AGGTGTGTGAAGAAGGAAGGAGATACTGGGGAAGGC R:GATAAGGTCCCTGGCTGTGGGGCTGCATGC	307	64 °C 30 s	35	*Hin*P1I
g.-911T>G (rs739858013)	F: CCTTATCTTGCCTGTTTACTC R: GCACTTGGACCTCGCTATT	379	55 °C 35 s	35	*Hin*FI
g.-891>T (rs735029013)	F: GGAGCCCTCTCTTCCCCTCTTCTT R:CGTGCTGTCTAAACGCTGTCCTGTA	332	61.8 °C 35 s	35	*Rsa*I
g.691C>T (rs16282937)	F: TCCACTGGTCCCCACTGTCCCGT R: GGTTGGGCACAAAGCCTCAAAAGC	273	57.8 °C 35 s	35	*Bst*NI
g.897T>C (rs316577617)	F: TCCACTGGTCCCCACTGTCCCGT R: GGTTGGGCACAAAGCCTCAAAAGC	273	60 °C 35 s	35	*Pvu*II
g.1033G>A (rs16282934)	F: GTCCCCTCCACTGGTCCCCACTGT R: GGGAGTGCTTTCTGGTGTGGCCG	394	60 °C 35 s	40	*Hin*P1I
g.1892A>G (rs793941727)	F:TTGTCTGAGACCTGTGGAATATGTAGATGCCTTGA R:GGCAATAATCCTCAGCCCCACACCGA	512	63.9 °C 30 s	35	*Bpu*EI
g.2091C>T (rs314263759)	F: TACTTTTCGTTTCCAACTCACCAGG R:GACATCTTGTAAACAGTGCGGTCATAAC	536	60 °C 30 s	35	*Ahd*I
g.2155C>T (rs16282929)	F:ATTGAGTGCGTGTGCAGTCGAGTGTC R:CTCAGAGTTGACCCTCCTTGGGGAGTC	432	62 °C 30 s	35	*Hpa*II

**Table 2:** The differences in allele frequencies of SNPs between the lean and fat lines.

SNPs	Strain	Genotype Frequency (No. of Birds)			Allele Frequency		χ^2^
g.-1243C>T		*CC*	*TC*	*TT*	*C*	*T*	
	Lean line	0.701 (235)	0.278 (93)	0.021 (7)	0.84	0.16	21.66577
	Fat line	0.547 (186)	0.379 (129)	0.074 (25)	0.737	0.263	(*p* < 0.01)
g.-1171T>C		*TT*	*TC*	*CC*	*T*	*C*	
	Lean line	0.728 (244)	0.266 (89)	0.006 (2)	0.861	0.139	28.45446
	Fat line	0.562 (190)	0.367 (124)	0.071 (24)	0.746	0.254	(*p* < 0.01)
g.-911T>G		*TT*	*TG*	*GG*	*T*	*G*	
	Lean line	0.697 (232)	0.294 (98)	0.009 (3)	0.844	0.156	20.67613
	Fat line	0.56 (190)	0.366 (124)	0.074 (25)	0.743	0.257	(*p* < 0.01)
g.-891C>T		*CC*	*CT*	*TT*	*C*	*T*	
	Lean line	0.77 (258)	0.227 (76)	0.003 (1)	0.884	0.116	39.40273
	Fat line	0.574 (195)	0.356 (121)	0.07 (24)	0.751	0.249	(*p* < 0.01)
g.691C>T		*CC*	*CT*	*TT*	*C*	*T*	
	Lean line	0.49 (164)	0.409 (137)	0.101 (34)	0.694	0.306	103.2983
	Fat line	0.2 (68)	0.438 (149)	0.362 (123)	0.419	0.581	(*p* < 0.01)
g.897T>C		*CC*	*CT*	*TT*	*G*	*T*	
	Lean line	0 (1)	0.3 (101)	0.7 (230)	0.155	0.845	133.2732
	Fat line	0.212 (72)	0.465 (158)	0.323 (110)	0.444	0.556	(*p* < 0.01)
g.1033G>A		*AA*	*AG*	*GG*	*A*	*G*	
	Lean line	0.62 (207)	0.32 (101)	0.06 (20)	0.78	0.22	69.4667
	Fat line	0.34 (116)	0.45 (151)	0.21 (71)	0.567	0.433	(*p* < 0.01)
g.1892A>G		*AA*	*AG*	*GG*	*A*	*G*	
	Lean line	0.184 (59)	0.763 (245)	0.053 (17)	0.565	0.435	38.83882
	Fat line	0.467 (154)	0.527 (174)	0.006 (2)	0.73	0.27	(*p* < 0.01)
g.2091C>T		*TT*	*TG*	*CC*	*T*	*C*	
	Lean line	0.8445 (277)	0.137 (45)	0.018 (6)	0.909	0.091	61.71857
	Fat line	1 (340)	0 (0)	0 (0)	1	0	(*p* < 0.01)
g.2155C>T		*TT*	*TC*	*CC*	*T*	*C*	
	Lean line	0.728 (244)	0.238 (80)	0.033 (11)	0.848	0.152	63.82669
	Fat line	0.45 (153)	0.421 (143)	0.129 (44)	0.66	0.34	(*p* < 0.01)

χ^2^ indicates chi-squared distribution.

**Table 3:** Phenotypic traits, heritability, and genetic correlation between abdominal fat and other phenotypic traits.

Traits (Unit)	Heritability	Genetic Correlation
AFW (g)	**0.485 ± 0.096**	1
BoL (cm)	0.069 ± 0.063	**−0.716 ± 0.308**
BW1 (g)	**0.500 ± 0.102**	**−0.332 ± 0.160**
BW3 (g)	**0.218 ± 0.080**	**−0.515 ± 0.194**
BW5 (g)	**0.304 ± 0.086**	**−0.345 ± 0.200**
BW7 (g)	**0.224 ± 0.080**	**−0.565 ± 0.190**
ChA (°)	0.096 ± 0.043	0.213 ± 0.132
ChW (cm)	0.083 ± 0.062	0.240 ± 0.306
CW (g)	**0.273 ± 0.091**	−0.155 ± 0.255
GSW (g)	**0.537 ± 0.097**	**−0.340 ± 0.150**
HW (g)	0.187 ± 0.072	0.165 ± 0.216
KeL (cm)	0.193 ± 0.073	**−0.329 ± 0.204**
LW (g)	0.131 ± 0.066	**0.696 ± 0.223**
MeC (cm)	**0.250 ± 0.084**	−0.133 ± 0.198
MeL (cm)	0.176 ± 0.076	**−0.438 ± 0.208**
MSW (g)	**0.662 ± 0.094**	−0.181 ± 0.145
TeW (g)	**0.413 ± 0.106**	0.199 ± 0.238

The bold indicates moderate or higher heritability or genetic correlation.

**Table 4:** Transcription factor binding site analysis.

SNPs	Base Group	Transcription Factors	Function
g.-1243C>T	C	BACH1	Myocardial ischemia [20]
g.-1171T>C	-	-	-
g.-911T>G	T	GATA4	Key regulators of heart gene expression [21,22]
		SMAD1	protects cardiomyocytes from ischemia-reperfusion injury [23]
		SOX17	Early heart development in mouse embryos [24,25]
g.-891C>T	-	-	-

## Data Availability

The datasets used and/or analyzed during the current study are available from the corresponding author on reasonable request.

## Supplementary Materials

The following supporting information can be downloaded at: https://www.mdpi.com/article/10.3390/ani12030393/s1, Figure S1: *TCF21* gene SNPs typing results.
