# Supplementary figures and images for: Associations of Transcription Factor 21 Gene Polymorphisms with the Growth and Body Composition Traits in Broilers
Source: Animals (Basel). 2022 Feb 8;12(3):393. doi: 10.3390/ani12030393 (PMC8833368; doi:10.3390/ani12030393)

Supplementary Materials:

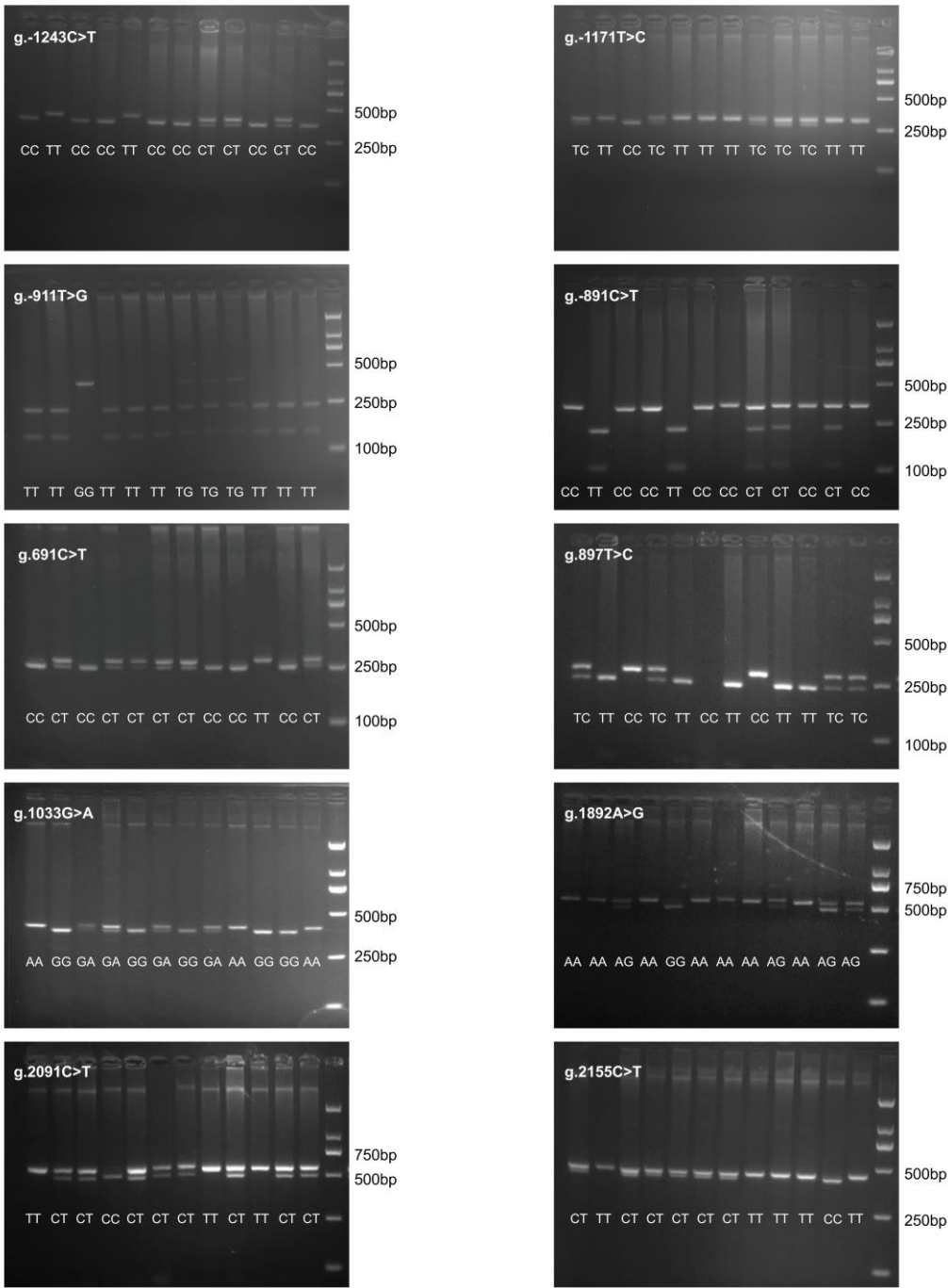

Figure S1: TCF21 gene SNPs typing results.

Supplement: Supplementary file 1 [file animals-12-00393-s001.zip › animals-1561582-supplementary.pdf]
